# Tramadol for treatment of neurogenic cough: a retrospective case series

**DOI:** 10.1017/S0022215125103915

**Published:** 2026-03

**Authors:** Benjamin Joseph Rubinstein, Sheela Saidha, John Todd Sinacori

**Affiliations:** Macon & Joan Brock Virginia Health Sciences Eastern Virginia Medical School, Old Dominion University, Norfolk, VA, USA

**Keywords:** chronic cough, cough hypersensitivity syndrome, neuromodulators, tramadol

## Abstract

**Objectives:**

This study aimed to explore the efficacy of tramadol for neurogenic cough and describe the longitudinal treatment experience.

**Methods:**

A retrospective case series of adults with chronic cough who were treated with tramadol for neurogenic cough. A complete response was defined as no pathologic coughing, and a partial response was defined as 50 per cent or greater reduction in severity or frequency.

**Results:**

Sixty-nine patients were included: 38 per cent of patients reported a complete response with an additional 33 per cent reporting a partial response. The most common successful dosing regimen was 50 mg twice a day. Age, gender and body mass index did not impact treatment response. Patients with a history of laryngeal surgery were less likely to respond (*p* = 0.04). Sedation (10 per cent) was the most common side effect. Fifty per cent of complete responders (*n* = 13) were weaned off tramadol after a mean of 39 weeks of treatment.

**Conclusion:**

Tramadol may be effective and is well tolerated in patients with neurogenic cough.

## Introduction

Chronic cough affects approximately 10 per cent of the population worldwide and causes significant morbidity.^1^ Expenditures on over-the-counter cough suppressant drugs amount to approximately $1 billion annually. The addition of prescription medications and clinical evaluation results in substantial healthcare spending.^2^ The defining feature, frequent cough, is associated with significant psychosocial issues and decreased quality of life.^3^

The aetiology of chronic cough is often attributed to inflammatory conditions such as asthma, gastroesophageal reflux disease (GERD) and allergic or infectious airway pathology. Investigations seeking an underlying cause are frequently extensive. A careful history and physical exam as well as selected diagnostic tests can be useful in determining the aetiology. In appropriately screened patients, a therapeutic trial of asthma, rhinosinusitis or acid reflux treatment can resolve the cough.^4^ Recent guidelines from the CHEST Expert Cough Panel also recommend optimising management of bronchial hyperresponsiveness and eosinophilic bronchitis when present.^5^

The role of neurogenic laryngeal hypersensitivity as a driver of chronic cough has been described in recent reviews detailing neurogenic cough^6^ and cough hypersensitivity syndrome (CHS).^7^ Classically, these patients have an irritation or ‘tickle’ sensation of the throat brought on by typically non-noxious stimuli (perfumes, cleaning products, cold air, talking), followed by a dry cough which is sometimes paroxysmal. Sensory receptors, pain neuropeptides and pain fibres in the airway mediate the cough reflex. The pain experience (throat tickle) and the behavioural output, a cough, are independent events for the patient that become tied together.^8^

Neuromodulator medications treat the hypersensitivity to benign stimuli, such as speaking and breathing.^6^ It is no coincidence that all of the medications used to treat chronic neurogenic cough are also used effectively for chronic pain. Recent studies have shown that patients with neurogenic cough may benefit from treatment with pregabalin, amitriptyline, nortriptyline and gabapentin.^9^^,^^10^ However, patients report significant side effects for some of the medications.^11^ The optimal pharmacologic treatment regimen for adults with chronic cough is still being investigated. An ideal neuromodulator for cough would treat both the sensory component and also be an effective central nervous system cough suppressant.

Tramadol is a medication that may be useful in the treatment of neurogenic cough because it has anti-tussive, analgesic and anti-nociceptive properties. Tramadol is an atypical opioid with a chemical structure very similar to venlafaxine, a serotonin norepinephrine reuptake inhibitor (SNRI), commonly used for anxiety, panic disorder and depression.^12^ Tramadol has the following pharmacologic properties: (1) μ opioid receptor agonist,^13^ (2) weak SNRI,^14^ (3) serotonin-2c receptor (5-HT_2c_) antagonist,^12^ (4) competitive inhibitor of muscarinic (M1 and M3) receptors,^15^^,^^16^ (5) non-competitive N-methyl-D-aspartate (NMDA) receptor antagonist^17^ and (6) complex agonist of the TRPV1 receptor.^16^^,^^18^^–^^20^ Indeed, a pilot study has suggested tramadol is effective in treating neurogenic cough.^21^ A recent survey of laryngologists in the United States indicated that 73 per cent prescribe tramadol for neurogenic cough, with 11 per cent using tramadol as first-line.^22^

This retrospective cohort study explores the use of tramadol for patients with neurogenic cough at a single institution. The aims of the study were two-fold. Firstly, to assess the response rates in those prescribed tramadol for neurogenic cough. Secondly, to assess the longitudinal treatment experience including typical dose, time to response and ability to wean the medication. The authors hypothesised that there would be a similar proportion of complete and partial cough resolution response rates as have been reported with other neuromodulators in the literature.

## Materials and methods

A retrospective chart review was performed for patients (ages 30–83) who were treated for neurogenic cough (ICD-9 code 786.2; ICD-10 code R05) over a 6.5-year period between January 2010 and August 2016. Institutional Review Board (IRB) approval was granted for retrospective chart review with waiver of consent and waiver of use of Protected Health Information (PHI). All patients were treated by a single laryngologist at an outpatient clinic in a tertiary academic medical centre. Patients were included if they were diagnosed with neurogenic cough by the academic laryngologist, had a chronic cough lasting greater than eight weeks and were treated with tramadol for neurogenic cough. Diagnosis of neurogenic cough was based on (1) classic history of pathologic coughing triggered by non-noxious stimuli, and (2) cough is unexplained by or refractory to guideline-based comprehensive workup and management of chronic cough. The responders and non-responders had the following rates of prior or concurrent trials of medication: proton pump inhibitors (PPIs) (80 per cent and 90 per cent), histamine-2 (H2) blockers (16 per cent and 5 per cent), inhaled corticosteroids (33 per cent and 35 per cent) and nasal corticosteroids (55 per cent vs. 40 per cent) (Table 1). Patients were excluded if they did not take the tramadol after receiving a prescription or if they did not have a follow-up appointment to determine efficacy.

**Table 1.** Characteristics of patients treated with tramadol for chronic cough

	Total *N* = 69	
		%
Age (average, years)	61 ± 13	
Sex		
Male	17	24%
Female	52	76%
Smoking history		
Former smoker	14	20%
Current smoker	2	3%
Body mass index (average, kg/m^2^)	29 ± 6	
Treatments used during history of coughing		
ACE inhibitor	3	4%
Proton pump inhibitor	57	83%
Histamine−2 blocker	9	13%
Inhaled corticosteroid	23	33%
Nasal steroid spray	35	50%
Continuous positive airway pressure	11	16%
Amitriptyline	11	16%
Gabapentin	14	20%
History of laryngotracheal surgery	11	16%

ACE = angiotensin-converting enzyme.

Patient demographics, tramadol doses and duration of therapy were recorded. Clinical responses were recorded from documentation on follow-up visits with laryngology. Patient reporting during clinic follow-up was used to assess for efficacy of treatment and assess complication rates. A complete response was defined as having no pathologic coughing reported by the patient at follow-up. A partial response was defined as at least 50 per cent reduction in frequency and/or severity of cough. Descriptive statistical analysis was performed to evaluate efficacy of tramadol treatment and frequency of side effects. Chi-squared analysis was performed to assess differences in response rates across clinical categories.

The senior author utilises tramadol as the first-line neuromodulator for neurogenic cough. The typical starting dose is tramadol 25 mg by mouth at bedtime with the following instructions provided to the patients. Patient are instructed to monitor for drowsiness the day following their first dose. Their first dose is to be taken on an evening that would allow them to avoid driving the next day if necessary due to drowsiness. If the patient tolerates the dose without unwanted side effects and has a complete response, they are to continue the starting dose. If they have too much drowsiness at any point, they are to lower the dose or discontinue the medication. If they have a partial response, they are instructed to increase the dose or frequency by changing to 25 mg twice a day or 50 mg at night. The patient can increase the dose independently up to 50 mg twice a day during the initial six-week trial period prior to their first clinic follow-up. First clinic follow-up is typically scheduled routinely as an office visit at four to eight weeks. Any further increase in dosing (greater than 50 mg per dose or up to three times daily) is done subsequent to the initial six weeks with guidance from the physician.

## Results and analysis

The mean age was 61 years (range 30–83; standard deviation [SD] = 13.6) and 76 per cent were female (Table 1). Of the 83 reviewed charts, five patients were excluded due to missing records; five due to lack of follow-up data; and four patients due to poor medication compliance. As such, 69 patients were studied. Mean follow-up was 18.6 months (range 1 to 75 months).

**Figure 1.**
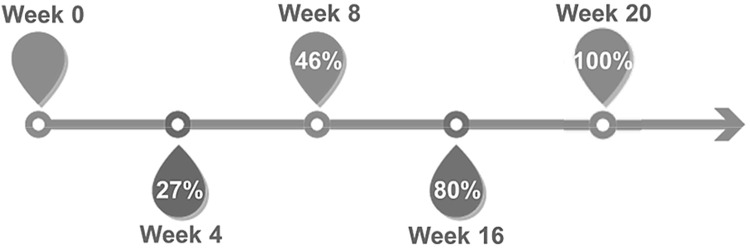
Monthly proportion of complete responders with cough cessation on tramadol.

A complete response (CR) was identified in 38 per cent of patients, a partial response (PR) in 33 per cent and no response (NR) in 29 per cent. Of the 26 patients achieving CR, 13 (50 per cent) successfully weaned off the tramadol after a mean of 9 months, 7 of 13 patients (50 per cent) with an initial complete response experienced recurrence of cough following tramadol weaning requiring re-initiation of treatment. These delayed relapses of chronic cough frequently followed an insult such as a new viral infection. Average total treatment time was 46 weeks for responders (median 25 weeks). Average total treatment time for complete responders who weaned off tramadol without relapse was 39 weeks (median 16 weeks). At last office visit, those patients still on tramadol were as follows: CR = 88 per cent; PR = 74per cent; NR = 25 per cent. The majority (80 per cent) of patients achieving a CR were asymptomatic by 16 weeks, and 27 per cent had stopped coughing within 4 weeks (Figure 1). There were seven patients who initially experienced a PR who ultimately developed a CR, most frequently due to continued medication trial at the same dose and in one patient due to dose escalation (from 50 mg to 100 mg twice daily). Of the NR group, 65 per cent used the medication no longer than eight weeks. Also, no patient who initially showed NR at first eight-week follow-up went on later develop a CR or PR (Table 2). There were only four patients in the NR group treated greater than 28 weeks; three who had a history of laryngeal surgery and were treated for 80, 160 and 160 weeks, respectively, and one patient treated for 44 weeks in the setting of attempted but unsuccessful dose escalation.

**Table 2.** Treatment duration and responses

	Response rate (% of total)	Average treatment duration (weeks)	Median treatment duration (weeks)	Successful wean No. (%)	Mean treatment duration if weaned without relapse (weeks)	Median treatment duration if weaned without relapse (weeks)	Taking tramadol at last office visit (%)
Complete responders	38%	60	25	13 (50%)	39	16	88%
Partial responders	33%	32	24	n/a	n/a	n/a	74%
Non-responders	29%	29	8	n/a	n/a	n/a	25%

**Figure 2.**
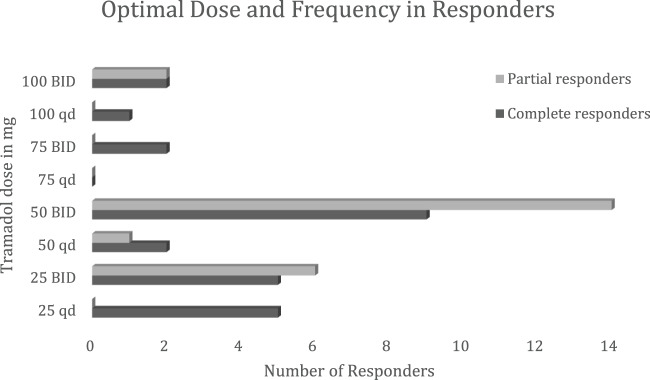
Optimal dose and frequency in complete and partial responders.

The most frequent successful tramadol dose was 50 mg twice daily. Figure 2 shows the frequency of final tramadol doses in the responders. On chi-squared analysis, patients with a history of laryngeal surgery (*p* = 0.04) were significantly less likely to respond to tramadol. Table 3 lists the prior laryngeal surgeries in complete responder, partial responder and non-responder groups. The response to tramadol was no different between sexes and was not associated with the cough being refractory to prior or current treatment trials for GERD, asthma or allergic rhinitis (Table 2). The response to tramadol was not different between smokers and non-smokers (*p* = 0.5). The use of other neuromodulators for chronic cough (gabapentin or amitriptyline) did not predict a response (*p* = 0.6). Speech language pathology therapy was utilised at some point during the treatment in 47 per cent of responders and 50 per cent of NR and did not modulate the response to tramadol (*p* = 0.8) (Table 4).

**Table 3.** Prior laryngeal surgeries in complete, partial and non-responders

Response group	Prior Laryngeal surgery	Otolaryngologic diagnoses
Complete responders	MDL with microflap excision of TVF cyst	TVF cyst, spasmodic dysphonia
Partial responders	Unilateral type 1 thyroplasty with silastic	Glomus jugulare
	MDL with laser excision and balloon dilation of SGS	Subglottic stenosis from prolonged intubation
	Left RLN resection and reinnervation with ansa graft	Papillary thyroid cancer
	MDL with microflap excision of TVF cyst	TVF cyst
Non-responders	MDL with laser excision and balloon dilation of SGS	Idiopathic subglottic stenosis
	UPPP, tracheotomy, pharyngoplasty, soft palate re-approximation and decannulation	OSA, VPI, nasopharyngeal stenosis
	MDL w/ microflap excision of vocal fold leukoplakia	Laryngeal leukoplakia, chronic laryngitis
	Injection laryngoplasty	Right TVF immobility after open heart surgery
	Left thyroarytenoid Botox injection	Laryngeal spasm
	MDL with excision of TVF polyps	TVF polyps

MDL = microdirect laryngoscopy; OSA = obstructive sleep apnoea; RLN = recurrent laryngeal nerve; SGS = subglottic stenosis; TVF = true vocal fold; UPPP = uvulopalatopharyngoplasty; VPI = velopharyngeal insufficiency.

**Table 4.** Association between cough improvement with tramadol and covariates

Parameter	Total *N* = 69		
	Responders	Non-responders	*p*-Value
	No. (%)	No. (%)	χ^2^ test
Gender			0.7
Female	37 (76%)	16 (70%)	
Male	12 (24%)	4 (30%)	
Reflux treatment*			0.8
Yes	43 (88%)	18 (90%)	
No	6 (12%)	2 (10%)	
Asthma*			0.9
Yes	14 (29%)	6 (30%)	
No	35 (71%)	14 (70%)	
Allergic rhinitis*			0.6
Yes	33 (67%)	12 (60%)	
No	16 (33%)	8 (40%)	
Laryngeal surgery			**0.04**
Yes	5 (10%)	6 (30%)	
No	44 (90%)	14 (70%)	
Radiation therapy			0.9
Yes	2 (3%)	1 (5%)	
No	47 (97%)	19 (95%)	
Smoker (current + former)			0.5
Yes	11 (22%)	6 (30%)	
No	38 (78%)	14 (70%)	
Other neuromodulators^†^			0.6
Yes	15 (31%)	5 (25%)	
No	34 (69%)	15 (75%)	
SLP intervention			0.8
Yes	23 (47%)	10 (50%)	
No	26 (53%)	10 (50%)	

* Denotes current or former treatment trials for these conditions.

† Denotes current or former treatment trials of gabapentin or amitriptyline.

SLP = speech language pathology.

Overall, 13 patients (18 per cent) experienced one or more side effects, with sedation being the most common (10 per cent). Six patients discontinued the medication due to side effects or drug interactions (two CRs, two PRs and two NRs). While five responders discontinued tramadol due to side effects, none of those patients experienced sedation. Rather, the reasons for discontinuing the medication included constipation, disorientation, nausea, itching and an unpleasant sensation. Two patients discontinued tramadol due to being prescribed either Effexor or Lexapro. One patient experienced insomnia but continued taking tramadol.

## Discussion

This retrospective review describes the use of tramadol for pharmacologic suppression of neurogenic cough over seven years at a single institution. The use of tramadol for chronic pain, as an alternative to typical opioid medications, has been previously described and advocated.^14^ The use of tramadol for neurogenic cough is not limited to our institution,^23^ but has been sparsely described in the literature. We present data suggest that tramadol is an effective and well tolerated neuromodulator in this setting.

The response rate for tramadol mirrors that described for other neuromodulators used in patients with chronic cough. In our study, CR was achieved in 38 per cent, a PR in 33 per cent and NR in 29 per cent. Stein and Noordzij described the use of amitriptyline in a cohort of 66 patients and described similar CR (32 per cent), PR (24 per cent) and NR (36 per cent) rates.^24^ Ryan and Cohen reviewed 36 patients and showed at least a 50 per cent reduction in cough in 67 per cent of patients at short-term follow-up and 53 per cent long-term.^11^ Song *et al*. described the use of nortriptyline in a cohort of 42 patients and also described similar rates of responders (72 per cent) and non-responders (28 per cent).^10^ Response rates for gabapentin parallel these results at 68 per cent.^25^ This similarity suggests that the medications are affecting a similar pathogenic process. The possibility exists that we are observing the natural history of the disease but that appears unlikely given that many of these patients come to attention following months of unsuccessful management. Also, randomised trials have proven a benefit for amitriptyline and gabapentin.^9^

Given the current concerns about the opioid epidemic,^26^ treatment duration and weaning rates are paramount. For CR, a quarter of patients were asymptomatic by four weeks and over three quarters by three months (Figure 1). The exact timing of response is limited by the retrospective nature of the study. Symptoms were measured on follow-up visits and frequently did not specify the exact response time. This timeline suggests, however, that attempts at weaning the medication could start after three to four months. Also, NR at eight weeks suggests no further medication trial is necessary after that point. A shorter course may be sufficient pending prospective studies. Once the common dose of 50 mg twice daily is reached, few patients will need further up-titrating. The abuse potential is considered low due to the pharmacodynamics of tramadol.^27^ An experience of “liking” tramadol occurs only at much higher doses.^28^ Appropriately, 75 per cent of non-responders independently discontinued the medication prior to their follow-up visit, and the other 25 per cent of non-responders discontinued with physician guidance.

Tramadol is practical as it is well tolerated and does not require complex dose titration. Dose adjustment is performed in 25 mg tablet increments. For patients with predominantly bedtime symptoms, nightly dosing is an option. Only 10 per cent of patients reported sedation, and interestingly no responder discontinued tramadol due to sedation. Only 6 per cent of responders discontinued tramadol and each secondary to a different complaint associated with its monoaminergic activity. This side effect profile is likely underestimated as no standardised questionnaire was provided to patients. Yet, higher rates of sedation (23 per cent)^24^ and discontinuation rates (32 per cent)^11^ are reported for amitriptyline. For nortriptyline, the rate of sedation is similar (21 per cent), and xerostomia is reported at 7 per cent.^10^ In a randomised trial of gabapentin, 31 per cent of patients had one or more adverse effects, most commonly dizziness, fatigue and abdominal symptoms.^29^ Twenty-five per cent of patients in a prospective evaluation of tramadol reported somnolence and management was accomplished with dose schedule adjustment.^21^ At our institution, patients are occasionally treated with gabapentin and amitriptyline, but tramadol is used as the first-line medication.

The use of opiates as a treatment for cough is not a novel concept, but tramadol appears to have pharmacologic characteristics making it ideal. Slow-release, low-dose morphine was evaluated in a randomised placebo-controlled trial and significantly improved cough scores and quality of life. A non-significant trend toward reduced objective cough reflex sensitivity on citric acid challenge testing was observed.^30^ The authors speculate that the effect of morphine is distinctly anti-tussive and note that other works have shown an objective decrease in cough reflex sensitivity with systemic opiates.^31^ Tramadol, unlike morphine, is an atypical opiate with non-morphine metabolites. In the animal model, both tramadol and its metabolite O-desmethyltramadol (M1) have agonistic effects on the μ opioid receptor. Tramadol alone does not account for the analgesic effects, as the M1 metabolite has a 300-fold greater affinity for the μ opioid receptor than tramadol. The affinity of M1 for the μ opioid receptor remains markedly lower than that of morphine.^13^^,^^32^ The fact that M1 is most active 2 hours after administration may account for the reduced experience of potency and thus abuse potential.

Tramadol may reduce chronic cough via the monoaminergic pathway as well. Tramadol is known to weakly inhibit reuptake of monoamines serotonin and norepinephrine, thus inhibiting nociception at the spinal level. Its structure is remarkably similar to venlafaxine, an SNRI, commonly used for anxiety, panic disorder and depression.^12^ It follows that other chronic pain medications acting on the monoamine system, such as amitriptyline or nortriptyline, would also provide benefit for chronic cough patients. Interestingly, tricyclic antidepressants are particularly useful for chronic pain relative to other antidepressants, and they have been recently shown to act weakly on opioid receptors.^33^

The muscarinic receptor antagonism of tramadol may alleviate airway reactivity and inflammation. Activation of the muscarinic receptor causes airway smooth muscle contraction and increased mucus secretion. There exists an emerging role of the muscarinic receptor in airway inflammation via cytokine release and lymphocyte proliferation. Increased vagal tone acting through the muscarinic pathway is one of the pathophysiologic conditions in asthma and chronic obstructive pulmonary disease (COPD) and is reversible with muscarinic blockade.^34^ Tramadol is a competitive inhibitor of muscarinic (M1 and M3) receptors at clinically relevant concentrations.^15^^,^^16^

To our knowledge, this study features the largest series of patients with chronic cough managed with tramadol. One of the main limitations of this study is the lack of a validated instrument to assess outcomes in this population of patients with chronic cough. In addition, due to the retrospective nature of the review we cannot comment on patient compliance with prescribed medications. This study was not designed to make any determination regarding the relative role of speech language pathology (SLP) treatment in chronic cough. An equal number of patients had participated in SLP in responders and non-responders.

Future prospective trials could study tramadol randomised against placebo, optimise weaning protocols and assess the specific central or peripheral effects of tramadol on the cough pathway. As with any anti-depressant or chronic pain medication, weaning off the medication is recommended for safety. Development of clinical protocols for determining treatment success and weaning of tramadol even in partial responders is likely warranted. The relative contributions of the various activities of tramadol might be elucidated with clinical trials including selective antagonism at the opioidergic, monoaminergic and muscarinic pathways. The utility of tramadol is likely due to synergistic effects which may have a relatively stronger role in any individual patient.

- Neuromodulator medications are routinely used to treat chronic neurogenic cough
- Tramadol is an atypical opioid structurally similar to venlafaxine, aserotonin norepinephrine reuptake inhibitor (SNRI)
- Tramadol is used as a neuromodulator for chronic cough, but there is less published research on its use when compared to other neuromodulators (gabapentin, amitriptyline and nortriptyline)
- In this study of patients with neurogenic cough, 38 per cent of patients reported a complete response with an additional 33 per cent reporting a partial response
- These response rates mirror those previously reported for gabapentin, amitriptyline and nortriptyline
- Tramadol was well tolerated in this series with simple, consistent dosing and rare discontinuation due to side effects
- A positive response to tramadol was uniformly evident by eight weeks, suggesting an extended trial of the medication is not necessary
- Fifty per cent of complete responders were weaned off tramadol successfully, though 50 per cent of those patients later experienced recurrent neurogenic cough, typically following a new respiratory illness, and restarted tramadol
